# Screening for Stereopsis of Children Using an Autostereoscopic Smartphone

**DOI:** 10.1155/2019/1570309

**Published:** 2019-10-31

**Authors:** Yanhui Yang, Huang Wu

**Affiliations:** ^1^Department of Pediatrics, Second Hospital of Jilin University, Changchun, China; ^2^Department of Optometry, Second Hospital of Jilin University, Changchun, China

## Abstract

**Background:**

The advantage of using an autostereoscopic smartphone is that it can achieve 3D effects without the need for glasses. The purpose of this study was to evaluate whether this technology could be utilized to detect stereoacuity.

**Methods:**

An autostereoscopic smartphone was used to imitate Lang stereotest I & II, Pass Test 3, Dinosaur Stereoacuity Test, and the Random Dot Stereo Acuity Test to screen the stereopsis of children from 3–6 years old.

**Results:**

No significant difference was found between each pair of groups (autostereoscopic smartphone vs. Lang stereotest I, Lang stereotest II, Pass Test 3, Dinosaur Stereoacuity Test, and Random Dot Stereo Acuity Test, respectively; Wilcoxon signed-rank test, *P* value all >0.05). All of the weighted kappa were higher than 0.84. Therefore, all of the comparisons between measurements showed a high level of agreement.

**Conclusions:**

The autostereoscopic smartphone is an effective tool when used for the screening of deficiency in stereopsis.

## 1. Background

Stereopsis is a kind of function by which subtle differences in distance can be judged precisely while stereoacuity is an index used to evaluate stereopsis. The principle for evaluating the threshold of stereoacuity is based on the minimum disparity that one can detect. Sometimes, testing is carried out in a real-life situation, such as in the Howard‒Dolman test [1], which is seldom used clinically nowadays, or the Frisby stereotest [2–4]. However, separating the two eyes is essential when testing for the threshold of disparity which is most commonly used in the clinical setting. Red- and green-colored spectacles are used in the TNO test [5–7], while polarized glasses are used in the Titmus test [8–10]. Computer-based testing is another powerful tool currently used for testing stereopsis, with polarized 3-dimensional (3D) technology [11] or 3D shutter glasses technology [12].

The development of naked-eye 3D technology has accelerated recently in the fields of advertising and entertainment, with its obvious advantage that glasses are no longer required [13–22]. Several techniques are used to turn a two-dimension (2D) picture into a 3D image without the aid of glasses. The essential aim of those methods was to transfer a 2D picture to one eye while transferring another 2D picture to another eye and then the predesigned disparities between the two 2D pictures would help to express a 3D image. Parallax barrier technology and lenticular technology are two mature techniques. The former contains vertical apertures to cover the light at certain angles to ensure sending different images to different eyes, while the latter uses the refraction function of microlenses to deviate the light to certain directions to different eyes [13]. However, some defects, e.g., the lower image resolution, reduced brightness, small viewing angles, and crosstalk, limited the application of the traditional method. Novel technologies have been created to address this problem, such as the use of the sub-pixel-level tunable lenticular liquid crystal (LC) lens array to obtain the same resolution as a 2D-display panel [17] or multidirectional backlight to provide a very efficient display [18].

Fujikado et al. [23] evaluated the stereopsis of strabismus patients using three-dimensional images displayed on a 10-inch LC display (resolution 640 × 480 pixels) equipped with an image-splitter system in almost 20 years ago. Breyer et al. [24] established a random-dot stereotest based on the use of an autostereoscopic monitor, and achieved a high correlation with the Lang stereotest I in children. However, the relatively low screen resolution of autostereoscopic tests renders these tests more useful as qualitative tools than precise quantitative tools.

**Figure 1:**
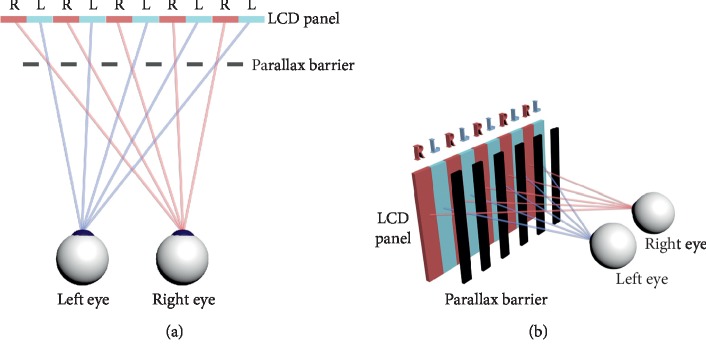
Diagram for parallax barrier autostereoscopic display. (a) Top-down view of eyes viewing. (b) 3D angle of viewing. Parallax barriers can send the right column to the right eye and the left column to the left eye, which means that what the right eye sees cannot be seen by the left eye and vice versa. Thus, binocular vision would be achieved when adding appropriate disparity elements between the two images.

The situation has been improved by the development of high-resolution smartphones with naked eye 3D technology. For a common naked eye 3D glasses-free mobile phone (e.g., ivvi K5 mobile phone [ivvi Scientific, NanChang, Co., Ltd. China]) equipped with a 5.5 inch IPS screen, the resolution of the display is 1920 × 1080 pixels. Parallax barrier technology was adopted to produce a 3D effect. The principle of the technology is schematically shown in Figure 1. The display density of the screen is 401 PPI (pixels per inch). At a checking distance of 40 cm, a pixel disparity equal to 33 seconds of arc (arcsec). Limited by the principle of parallax barrier technology (Figure 1), the minimum disparity would be twice of the physical pixels of the screen. Therefore, the test threshold was 66″ at 40 cm. When the distance was prolonged to 65 cm, the test threshold would approach 40″. However, the stereopsis threshold value may not be precise enough to measure a people's stereoacuity, it is usable to be a screen tool. For example, the threshold of Lang stereotest I & II (Lang-Stereotest AG, Kusnacht, Switzerland) is 550″ and 200″, respectively; the threshold of PASS Test 3 (Vision Assessment Corporation, Illinois, USA) and Dinosaur Stereoacuity Test (Bernell, a Division of Vision Training Products, inc. Indiana, USA) is 60″ and 40″, respectively.

We were interested in investigating the effect of this technology utilized to detect stereoacuity with a portable autostereoscopic smartphone, utilizing its flexibility with the aim of obviating the need for the wearing of glasses.

## 2. Methods

### 2.1. Subjects

The study was conducted at the Second Hospital of Jilin University in China. A total of 51 children were enrolled, comprising 30 boys and 21 girls, aged 3–6 (4.6 ± 1.0) years. Before participation in the study, informed consent was obtained from the guardian for all underage participants. The research protocol followed the tenets of the Declaration of Helsinki and was approved by the ethics committee of the Second Hospital of Jilin University (No. 2017–89).

### 2.2. Smartphone and Comparison Tests

**Figure 2:**
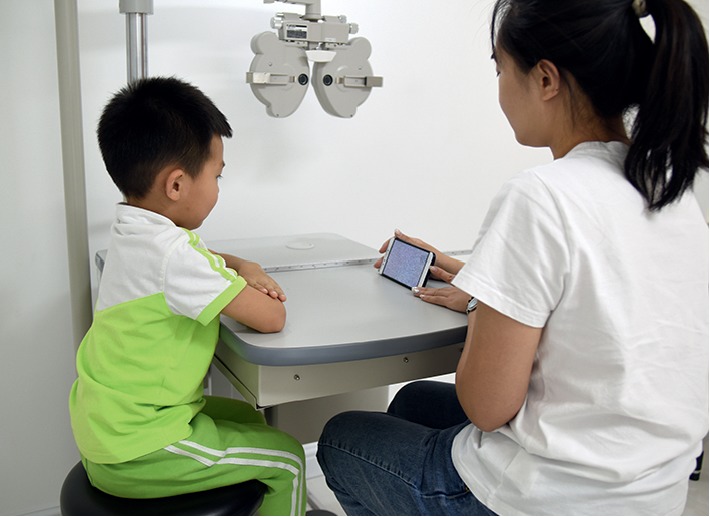
Photograph of the actual testing.

Naked eye 3D glasses-free original ivvi K5 mobile phone (display resolution: 1920 × 1080 pixels, display density: 401PPI) was used as a stereopsis evaluation tool. Actual test picture is shown in Figure 2. Five stereotests, Lang stereotest I & II, PASS Test 3, Dinosaur Stereoacuity Test and Random-Dot Stereo Acuity Test (Vision Assessment Corporation, Illinois, USA), were chosen as Screening Stereo tests.

### 2.3. Test Targets Design and Test Methods

#### 2.3.1. Imitating Lang Stereotest I & II

**Figure 3:**
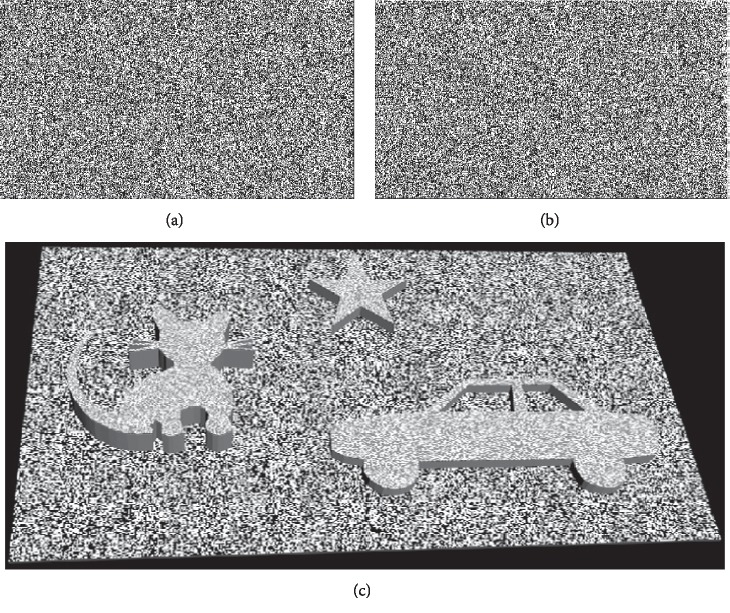
Imitating Lang stereotest I. (a) The picture viewed by the left eye. (b) The picture viewed by the right eye. (c) Simulation of the percepts generated by the test images. The pattern of cat, star, and car appear to pop out of the background plane. The disparity of the cat, star, and car was 36 pixels (equivalent to 1200″), 18 pixels (equivalent to 600″), and 16 pixels (equivalent to 550″), respectively.

A program written in C# was used to produce random-dot test targets (Figure 3). The test method was the same as the test procedure as standard Lang stereotest. The test distance was 40 cm. The disparity of the cat, star, and car in Lang stereotest I was 36 pixels (equivalent to 1200″ at 40 cm, similarly hereinafter), 18 pixels (600″), and 16 pixels (550″) (Figure 3). The disparities of the elephant, truck, moon, and star in Lang stereotest II were 18 pixels (600″), 12 pixels (400″), 6 pixels (200″), and 6 pixels (200″), respectively.

#### 2.3.2. Imitating Pass Test 3

**Figure 4:**
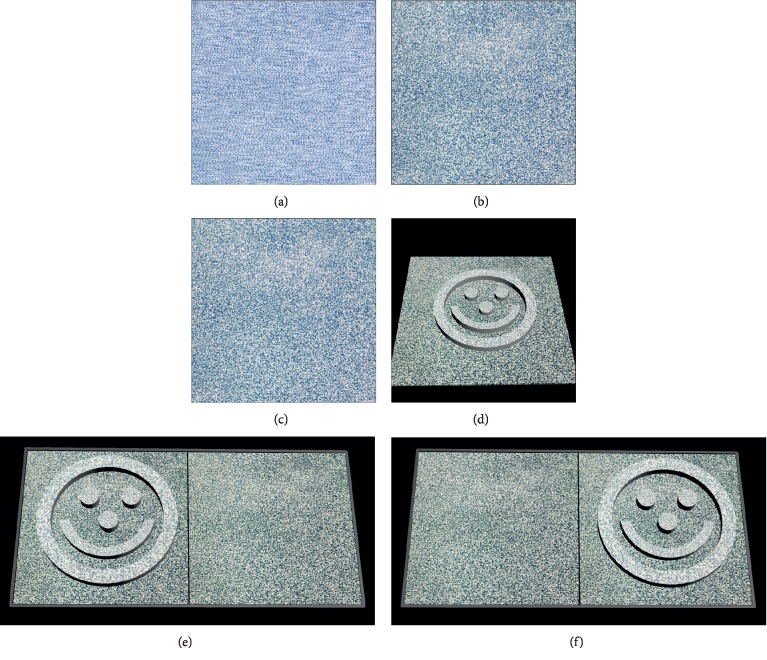
Legend to imitate pass test 3. (a) Card B (480″) was scanned at a normal pattern. The picture was blurred. (b) Picture B was scanned with the help of a polarizer film and was seen only by the left eye when wearing polarizing glasses at exam mode. (c) Picture C was scanned with the help of a polarizer film with the polarization direction perpendicular to picture B and was seen only by the right eye when wearing polarizing glasses at exam mode. (d) When fusing picture B and (C) a smile may appear out of the plane. (e) Position 1. The left side was the target side (960 × 1080) while the right part was blank comparison (960 × 1080). (f) Position 2, the right side was the target side while the left part was blank comparison. Positing 1 and 2 could be changed by the examiner to simulate the position of target and blank card as tested in the original test.

A scanner (ScanMaker S260, Microtek International, Inc. Shanghai, China) was used to scan the card. A polarizer film was covered on the surface of the card when scanning, while the position should be aligned with the polarization direction of the drawing. After two scanning with the help of polarizer film, a blur picture would be decomposed into two clear pictures (Figure 4). Adjust the pictures to 401PPI and cut the surrounding part to a size of 960 × 1080 pixels. For card B (480″), C (240″), D (120″), and E (60″), the disparities of the two pictures were 16 pixels, 8 pixels, 4 pixels and 2 pixels, respectively when setting the checking distance at 43 cm. The original pictures were used to do the test. The sample of the pictures is shown in Figure 2.

For the original test, examiner should hold the test card and blank card side by side and ask the subject to choose at which side of the card containing smile face. The position should be changed several times in the following tests and ask the subject to do the choice. In the test of a smartphone, each test disparity has two pictures (Figure 4). Examiner should choose which picture to be expressed randomly and let the subjects to point out which side contains smile face. During the test procedure, the head of the subject should not swap. The smile face may come out of the plane (crossed disparity) or go inside the plane (uncrossed disparity).

#### 2.3.3. Imitating Dinosaur Stereoacuity Test

**Figure 5:**
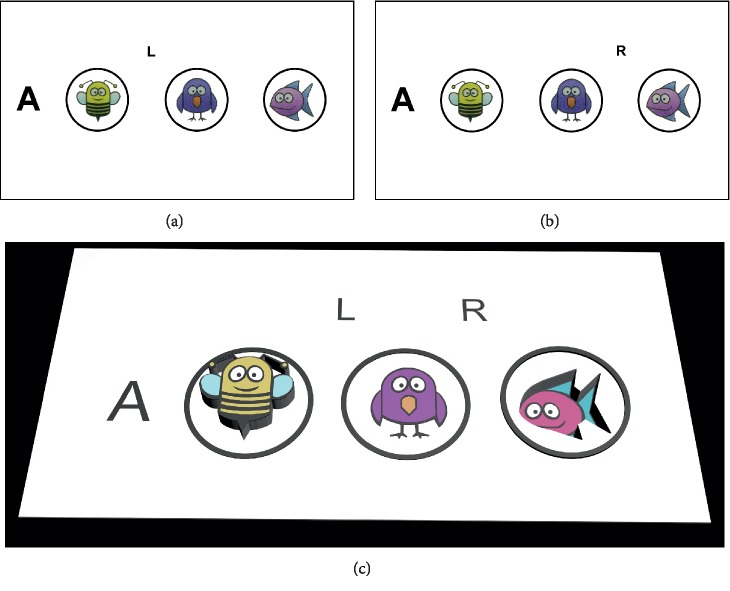
Legend of part 2 line A of dinosaur stereoacuity test. (a) The picture viewed by the left eye. (b) The picture viewed by the right eye. (c) Simulation of the percepts generated by the test images. At a correct watching condition, the right eye would see letter “R” and could not see letter “L”; meanwhile the left eye could only see letter “L” without seeing letter “R”. In line A, “bee” would appear out of the plane (12 pixels, equivalent to 400″), while the fish appear dent into the plane (12 pixels, equivalent to 400″).

Imitating part 2 of the Dinosaur Stereoacuity Test, which including 400″, 200″, and 80″. The animal may stand out of the plane (crossed disparity) or dent into the plane (uncrossed disparity) (Figure 5). The test distance was 40 cm, at which 12 pixels and 6 pixels disparities were equivalent to 400″ and 200″, respectively. When the distance was changed to 33 cm, 2 pixels were equivalent to 80″.

#### 2.3.4. Imitating Random Dot Stereo Acuity Test

**Figure 6:**
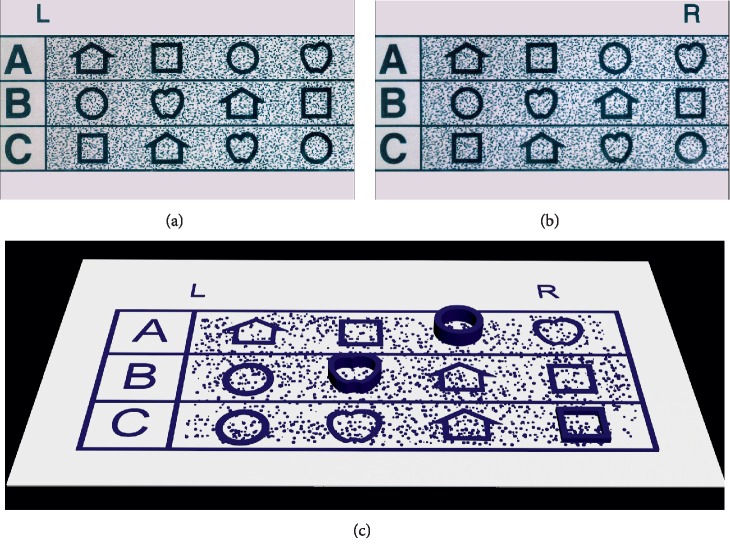
Legend of random dot stereo acuity test part 3. (a) The picture viewed by the left eye. (b) The picture viewed by the right eye. (c) Simulation of the percepts generated by the test images. At a correct watching condition, the right eye would see letter “R” and could not see letter “L”; meanwhile, the left eye could only see letter “L” without seeing letter “R”. In line A, “circle” would appear out of the plane (16 px, equivalent to 400″). In line B, “apple” would appear out of the plane (8 px, equivalent to 200″). In line C, “square” would appear out of the plane (4 px, equivalent to 100″).

Imitating Part 3 of Random Dot Stereo Acuity Test, which including 400″, 200″ and 100″ (Figure 6). The test distance was 50 cm, at which 16 pixels and 8 pixels and 4 pixels disparities were approximately equal to 400″, 200″ and 100″, respectively. The target symbol may appear standing out of or denting into the plane.

### 2.4. Statistical Analysis

Wilcoxon signed-rank test was used to explore the difference between groups (PASW Statistics 18 software [IBM SPSS Inc. Illinois, USA]). The weighted kappa method was used to evaluate the agreement between the two tests (MedCalc Statistical Software [version 17.6, MedCalc Software bvba, Ostend, Belgium]).

## 3. Results

The comparative data of the children between autostereoscopic smartphone and Lang stereotest I (Table 1), Lang stereotest II (Table 2), Pass Test 3 (Table 3), Dinosaur Stereoacuity Test (Table 4), and the Random Dot Stereo Acuity Test (Table 5) are shown in Table 6. Two children refused to do Pass Test 3 and the Random Dot Stereo Acuity Test; three children refused to do Dinosaur Stereoacuity Test. No significant difference was found between each pair of groups (autostereoscopic smartphone vs. Lang stereotest I; autostereoscopic smartphone vs. Lang stereotest II; autostereoscopic smartphone vs. Pass Test 3; autostereoscopic smartphone vs. Dinosaur Stereoacuity Test; autostereoscopic smartphone vs. Random Dot Stereo Acuity Test, and Wilcoxon signed-rank test, *P* value all >0.05, Table 6). All of the weighted kappa were higher than 0.84, and all of the lower limit of 95% confidence interval of weighted kappa were higher than 0.70 (Table 6). Therefore, all of the comparisons between the measurements showed a high level of agreement according to the Kappa Statistic (kappa in the range 0.61–0.80 shows substantial agreement; 0.81–0.99 shows almost perfect agreement [25]).

## 4. Discussion

The methods available to evaluate stereopsis are varied, from the Howard‒Dolman test, which was introduced over a100 years ago, to the most recent computer-aided 3D technology [11, 12, 26]. The chief techniques currently used to achieve computerized 3D effects are polarization and active LC shutter glasses technology.

Handheld mobile terminals, tablets or smartphones, have been used as effective instruments to evaluate stereopsis in recent years. IPad application was used to evaluate stereopsis at multiple distance. Rodríguez-Vallejo et al. presented a new stereoacuity test, called TST, performed on an iPad (2048-by-1536-pixel resolution and 264 ppi) [27]. The identified mission was almost the same with the TNO test. Anaglyph spectacles were used to watch test patterns with red and cyan colors displayed on the screen. The task for the observer is to identify the position of a missing section of a circle that appears at one of four possible orientations. Bonfanti et al. presented an android application called “Stereo Acuity Test” [28]. A smartphone was inserted into a Google Cardboard. The smartphone screen was split into two parts with the help of two lenses installed inside Google Cardboard. Then the images displayed in two parts of the screen were sent into the two eyes respectively. Random dot images were utilized to test stereopsis. The points inside the specific shape were horizontally shifted by a desired number of pixel between the images sent to the right and the left eye. A stereo vision would be produced by the shifting of specific parts. We have also done some research work on stereopsis with two 4K smartphones [26, 29, 30]. The display of a 4K mobile phone can produce a disparity small enough to measure the stereoacuity at a relatively short distance. A plastic sheet was attached to the near vision rod of a phoropter to separate the two eyes completely. All of these stereopsis measurement with the aid of handheld mobile terminals showed satisfactory results. However, additional instrument, such as anaglyph spectacles, Google Cardboard, or a phoropter, should be utilized to separate eyes.

The autostereoscopic method, or naked eye 3D display, is currently used mainly for large-screen displays in advertising or home entertainment. Glassless 3D technology used in smartphones has now become reality. The advantage of this technique is that the 3D effect could be observed without any other accessories. It is not known thus far whether this would become an effective method for the evaluation of stereopsis.

Theoretically, autostereoscopic technology should separate the image when reaching eyes, which means that what the right eye sees cannot be seen by the left eye and vice versa, as occurs when using polarization or the active LC shutter glasses technique to separate binocular images with spectacles. But, some researchers have done remarkable work to reduce the crosstalk between the left and the right eyes' images presented on digital autostereoscopic displays [31].

Inducing autostereoscopic technology to smartphone was helping to meet the demand of playing 3D games or watching 3D movies without wearing additional spectacles. Because of the limitation of the size of screen, the smartphone equipped with naked 3D technique was not that popular as initial conception. However, the small size and high resolution of the display of the smartphone provides fine enough dot pitch to achieve disparity practically to check stereopsis in a relatively near distance. The ivvi K5 mobile phone used in this experiment (490 PPI) can measure 40″ with a 65-cm checking distance. The precision achieved by the smartphone meets the accuracy of some commonly used stereopsis screening tools in the clinic.

Lang stereotest and Dinosaur Stereoacuity Test all belong to glass-free stereopsis screening tools. Dinosaur Stereoacuity Test is also a very special tool because of chromatic elements added into the test symbols. Pass Test 3 and Random Dot Stereo Acuity Test are all performed with the assistance of polarizing glasses. It is not complicated to imitate those test materials with an autostereoscopic smartphone. Based on our test result, evaluating stereopsis with an autostereoscopic smartphones and the examination tools showed a high level of agreement. Therefore, utilizing the autostereoscopic smartphone to evaluate stereopsis is feasible as a screening tool. The characteristic of the naked eye 3D smartphone, as being convenient to carry, easy to interact, simple to operate, flexible to create, etc., may give more choices for researchers or doctors to evaluate stereopsis in clinical or research field.

A further problem is caused by autostereoscopy per se, which does not occur with polarized or active shutter glasses 3D technology. It remains an unresolved issue between crossed and uncrossed disparity. Lenticular arrays or parallax barriers can send one image to one eye and another image to the other eye, thus achieving binocular vision. However, the technology itself cannot determine which image is transmitted to which eye, and the difference may be due to the discrepancies between individuals in the pupillary distance, watching distance, or viewing angle. At first glance, the test symbol may appear nearer than the others when crossed disparity appears, while it can also appear further than the others in the presence of uncrossed disparity. Fortunately, the reorganization depends on the amount of disparity, while it was not affected by the type of disparity. Whatever the stereo target appeared to recede into the screen or more prominent than the others, the target symbols can be distinguished correctly when the threshold of stereopsis was better than the testing disparity of a subject. However, false-positive may appear when the test booklets or the head of the subject moved during the test. The stereo target will appear reversing when the head is moved without stereopsis or when covering one eye, and the special symbols could readily be identified especially in images with relatively large disparity. It is important to keep the test booklets and the head of the subject stationary when starting to test.

With the development of information technology, smartphone has been widely utilized in more and more fields. Lots of apps for various purposes was created in ophthalmology expanding the application of smartphone [32]. As an intelligent terminal with high-resolution and high-brightness screen, visual acuity [33], color vision [34], and contrast sensitivity [35] could be tested precisely. As a high performance computer, it can serve to calculate IOL power [36], refractive error [37], or the grading of diabetic retinopathy [38]. With the help of the high-resolution built-in camera, it could be used as a slit-lamp to check eyelid [39], corneal [40], iris [41], etc., and as a fundus photography to examine retina [42]. As an intelligent interactive instrument, it can be used to assist eye exercising, such as amblyopia treatment [43].

Limitation of our experiment is that the test is just to imitate the commercially available stereopsis evaluation tools. Not an app was established to create a new test pattern. All of the tests were carried out for a relatively normal population. Extensive testing of defective vision subjects should be conducted to evaluate the method comprehensively.

## 5. Conclusions

With the recent development of computer-aided 3D technology, increasingly greater numbers of innovative methods relying on this technology for the evaluation of stereopsis could become a reality. The autostereoscopic smartphone can be used as an effective tool for evaluating deficiency in stereopsis.

## Figures and Tables

**Table 1:** Comparative data of autostereoscopic smartphone vs. Lang stereotest I (*n* = 51).

		Autostereoscopic smartphone			
		>1200″	1200″	600″	550″
Lang stereotest I	>1200″	1	0	0	0
	1200″	0	1	0	0
	600″	0	0	3	2
	550″	0	0	1	43

**Table 2:** Comparative data of autostereoscopic smartphone vs. Lang stereotest II (*n* = 51).

		Autostereoscopic smartphone			
		>600″	600″	400″	200″
Lang stereotest II	>600″	1	0	0	0
	600″	0	1	0	0
	400″	0	0	3	3
	200″	0	0	1	42

**Table 3:** Comparative data of autostereoscopic smartphone vs. Pass test 3 (*n* = 49).

		Autostereoscopic smartphone				
		>480″	480″	240″	120″	60″
Pass Test 3	>480″	1	0	0	0	0
	480″	0	1	0	0	0
	240″	0	1	0	1	0
	120″	0	0	1	6	3
	60″	0	0	0	0	35

**Table 4:** Comparative data of autostereoscopic smartphone vs. Dinosaur Stereoacuity Test (*n* = 48).

		Autostereoscopic smartphone			
		>400″	400″	200″	80″
Dinosaur Stereoacuity Test	>400″	4	0	0	0
	400″	0	5	2	0
	200″	0	0	4	2
	80″	0	1	7	23

**Table 5:** Comparative data of autostereoscopic smartphone vs. Random Dot Stereo Acuity Test (*n* = 49).

		Autostereoscopic smartphone			
		>400″	400″	200″	100″
Random Dot Stereo Acuity Test	>400″	3	1	0	0
	400″	1	2	0	0
	200″	1	0	9	5
	100″	0	0	2	25

**Table 6:** Comparative result between groups.

Comparison stereotest	Autostereoscopic smartphone			
	Wilcoxon signed ranks test		Interrater agreement (kappa)	
	Z	*P*	The quadratic weighted kappa	95% confidence interval
Lang stereotest I	−0.577	0.564	0.905	0.761 to 1.000
Lang stereotest II	−1.000	0.317	0.875	0.704 to 1.000
Pass Test 3	−0.816	0.414	0.916	0.830 to 1.000
Dinosaur Stereoacuity Test	−1.291	0.197	0.840	0.717 to 0.962
Random Dot Stereo Acuity Test	−0.277	0.782	0.852	0.741 to 0.963

## Data Availability

All the raw data of this article are shown in Supplementary table. The data of personal identity information will not be made available in order to protect the participants' privacy.

## Supplementary Materials

Supplementary Materials Test results of the children between autostereoscopic smartphone and Lang stereotest I, Lang stereotest II, Pass Test 3, Dinosaur Stereoacuity Test, and the Random Dot Stereo Acuity Test.
